# Comparative Analysis of the Simian Varicella Virus and Varicella Zoster Virus Genomes

**DOI:** 10.3390/v14050844

**Published:** 2022-04-19

**Authors:** Wayne L. Gray

**Affiliations:** Biology Department, University of Mississippi, Oxford, MS 38677, USA; wlgray@olemiss.edu

**Keywords:** varicella zoster virus, simian varicella virus, herpesvirus

## Abstract

Varicella zoster virus (VZV) and simian varicella virus (SVV) cause varicella (chickenpox) in children and nonhuman primates, respectively. After resolution of acute disease, the viruses establish latent infection in neural ganglia, after which they may reactivate to cause a secondary disease, such as herpes zoster. SVV infection of nonhuman primates provides a model to investigate VZV pathogenesis and antiviral strategies. The VZV and SVV genomes are similar in size and structure and share 70–75% DNA homology. SVV and VZV DNAs are co-linear in gene arrangement with the exception of the left end of the viral genomes. Viral gene expression is regulated into immediate early, early, and late transcription during in vitro and in vivo infection. During viral latency, VZV and SVV gene expression is limited to transcription of a viral latency-associated transcript (VLT). VZV and SVV are closely related alphaherpesviruses that likely arose from an ancestral varicella virus that evolved through cospeciation into species-specific viruses.

## 1. Introduction

Varicella zoster virus (VZV) and simian varicella virus (SVV) are herpesviruses that cause erythematous diseases in humans and Old World monkeys, respectively. VZV (*Human herpesvirus 3*) produces varicella (chickenpox) in children, characterized by vesicular skin lesions over the entire body [1]. After resolution of the primary disease, VZV establishes latent infection in neural ganglia throughout the neuraxis. Subsequently, the virus may be induced to reactivate to cause herpes zoster (shingles) with vesicular skin lesions associated with one or a few dermatomes [1].

SVV (*Cercopithecine herpesvirus 9*) causes a varicella-like disease in cercopithecoid monkeys, including patas and vervet monkeys, and several macaque species [2]. Infected monkeys exhibit fever, malaise, and a whole-body vesicular skin rash. Like VZV, SVV establishes latent infection in neural ganglia [3]. Stress or immunosuppression may induce viral reactivation although unlike VZV and for unclear reasons, SVV reactivation disease may be systemic and not associated with dermatomal spread [4]. Sporadic epizootics of simian varicella in facilities housing nonhuman primates have sometimes been associated with severe disease with high morbidity and mortality, while other outbreaks have exhibited milder disease [2,5].

Studies of VZV pathogenesis and development and evaluation of antiviral strategies are limited since VZV is exclusively a human virus. Based on the clinical and pathological similarities of simian and human varicella, SVV infection of nonhuman primates is a useful experimental model to study mechanisms of VZV pathogenesis and to develop and evaluate antiviral chemotherapies and vaccines [2].

SVV and VZV are species-specific herpesviruses. SVV replicates optimally in cell cultures of simian origin, such as African green monkey (Vero) cells, while VZV grows best in human cell culture, such as melanoma (Mewo) cells. Experimental VZV infection does not induce a varicella-like illness in nonhuman primates. Likewise, there are no reported cases of SVV causing disease in humans.

SVV and VZV are morphologically similar herpesviruses with an icosahedral capsid enclosing the viral double-stranded DNA genomes and surrounded by a viral envelope. The viruses are antigenically related. Immune serum from SVV infected monkeys immunoprecipitates cross-reacting VZV antigens and also neutralizes VZV in plaque-reduction neutralization tests (PRNT) [6,7]. Conversely, VZV immune serum from zoster patients immunoprecipitates SVV antigens and neutralizes SVV. In addition, experimental immunization of nonhuman primates with VZV induces immune protection against simian varicella following SVV challenge [8].

SVV and VZV also share extensive genetic relatedness. While the viral DNAs have distinct restriction endonuclease profiles, they share 70–75% DNA homology as determined by DNA hybridization assays using conditions of varying stringency as well as DNA sequence analysis [9,10]. Considering the close antigenic and genetic relatedness between SVV and VZV, the basis of the species-specificity of the viruses is unclear. This review compares the genetic characteristics of SVV and VZV genomes, their gene expression, and their evolutionary relationship.

## 2. Size, Genetic Content, and Structure of the SVV and VZV Genomes

The SVV and VZV genomes are the smallest in size among the alphaherpesviruses. The complete DNA sequence of the VZV prototype Dumas strain is 124,884 base pairs (bp) in length [11] although the DNA sequences of other VZV isolates vary with a size range of 124,770 to 125,945 bp. The size of the SVV genome is slightly smaller with 124,785 bp (deltaherpesvirus isolate) [10].

The guanosine + cytosine (G + C) content of SVV DNA was determined to be 40.8% by buoyant density analysis on cesium chloride gradients and confirmed to be 40.4% by DNA sequence analysis [10,12]. The VZV genome has a slightly higher G + C % content of 46% [11].

SVV and VZV DNA exist in an extrachromosomal configuration in infected cells. The viral DNAs may be in a circular form as shown by PCR utilizing primers from the termini of the viral genomes [13,14]. An unpaired 3’ nucleotide at the end of viral DNA may facilitate DNA circulation. The viral DNAs may also exist in concatameric head-to-tail forms, which are generated through the rolling circle mechanism of DNA replication as occurs in herpesvirus DNA synthesis [13]. SVV and VZV DNA also exist in a circular, extrachromosomal form in neurons of latently infected ganglia.

Consistent with other herpesviruses, the SVV and VZV genomes include direct and inverted repeat sequences [11,15]. Electron microscopy of denatured and then re-annealed SVV DNA revealed molecular structures consisting of a S 7.2 kb double-stranded stem with a ≈ 5.2 kb single-stranded loop [16]. This stem and loop structure was linked to a ≈ 100 bp single-stranded DNA stretch. Subsequent DNA sequence analysis confirmed that SVV DNA consists of a 20 kb short (S) component that includes a 5.2 kb unique (US) sequence bracketed by 7.5 kp internal and terminal inverted repeat (IRS and TRS) sequences [10]. This S component is covalently linked to a 104.7 kb long (L) component, which includes a 104.0 kb unique long sequence (UL) bracketed by 65 bp inverted repeat sequences (TRL and IRL).

**Figure 1:**
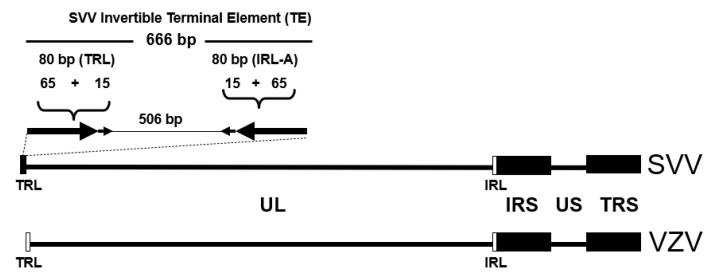
The SVV and VZV genomes consist of a short (S) component linked to a long (L) component. The S includes a unique sequence (US) flanked by internal and terminal inverted repeats (IRS, TRS). The L component includes a unique long (UL) segment bracketed by internal and terminal repeats (IRL, TRL). The SVV genome has an invertible terminal element (TE) that does not exist in VZV DNA.

This structure of SVV DNA is similar to the organization of the VZV genome (Figure 1, Table 1). The sizes of the various components of the SVV and VZV genomes are comparable. For example, the inverted repeat sequences of SVV DNA S component (TRS/IRS) at 7557 bp are slightly longer than that found in the VZV genome by 238 bp, while the SVV US sequence is 328 bp shorter than the VZV US.

The S and the L components of the SVV and VZV genomes can invert relative to each other, permitting the viral genomes to exist in two equimolar isomeric forms. This finding is based upon the existence of 0.5 molar DNA fragments in the restriction endonuclease profiles of the DNAs [16].

The SVV and VZV genomes include tandem direct repeat sequences [10,11]. SVV DNA has four of these repeat elements, R1, R2, R3, and R4, which are distributed along the SVV genome (Figure 1). The R1, located between ORFs 7 and 8, includes an A + T-rich 37 bp sequence that is repeated three times. The R2 present within the glycoprotein C gene (ORF 14) consists of two G + C-rich 83 bp sequences with an intervening 13 bp sequence. The R3 is the largest and most complex repeat element comprising seventeen 9 bp sequences and eight 12 base sequences and is within ORF 22. The R4 is a 16 bp G + C-rich sequence located between ORFs 62 and 63. VZV DNA also has repeat elements that correspond in location with the SVV R2, R3, and R4 although they vary in length and sequence composition [11]. The VZV genome has an R1 element that does not correspond in location with the SVV R1 but rather is positioned within VZV ORF 11. While direct repeat elements are conserved within herpesvirus genomes, their function is not understood.

The left terminus of the SVV and VZV genomes includes the most structural diversity between the viral DNAs. The left end of SVV genome includes a 666 bp terminal element that is not present within VZV DNA (Figure 1) [17]. This terminal element includes a 506 bp unique sequence surrounded by two 80 bp inverted repeat sequences. The inverted repeat sequences include the 65 bp IRL and TRL inverted repeats that bracket the UL component of SVV DNA. The terminal element is conserved among SVV isolates but may vary in length since some SVV isolates have an additional 46 bp repeat within the unique sequence. While VZV DNA does not contain a comparable terminal element, this component is found in the genomes of other *Varicelloviruses*, including pseudorabies herpesvirus (PRV) and equine herpesviruses types 1 and 4 (EHV-1, EHV-4) [17].

## 3. Gene Organization of the SVV and VZV Genomes

**Figure 2:**
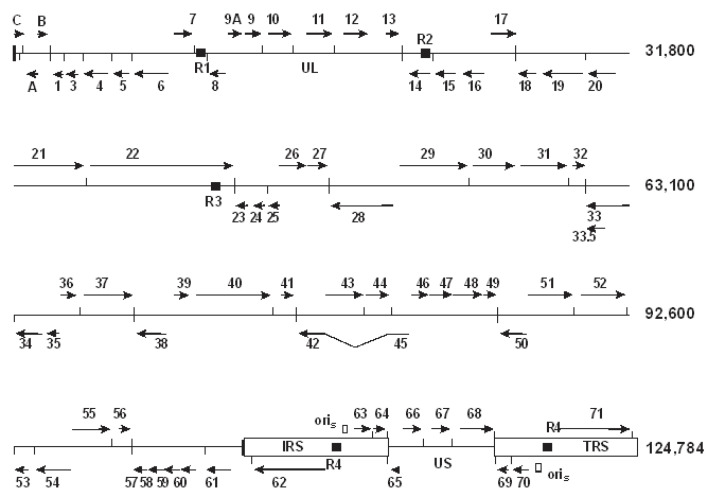
The SVV genome includes 73 ORFs, each of which are designated as arrows indicating the site on each DNA strand. The R1, R2, R3, and R4 direct repeats are indicated as boxes. The VZV genome has a similar gene map except for the left end of the viral DNA. VZV ORFs have the same nomenclature as their SVV homologs, while unique SVV ORFs are designated with letters (A, B, C). Vertical lines indicate poly A sites. Reprinted from Current Topics in Microbiology and Immunology [15] with permission from the publisher.

The SVV genome encodes 73 identified open reading frames (ORFs), of which three (ORFs 69, 70, and 71) are duplicated within the IRS and TRS (Figure 2). VZV DNA includes 72 ORFs with the same three duplicated genes. Viral ORFs along the SVV and VZV genomes are co-linear with respect to gene location. With only two exceptions, each SVV gene has a corresponding VZV homologue (Table S1). The homology based upon predicted amino acid sequence ranges from 75% for glycoprotein B (ORF 31) to 27% for ORF 1.

**Figure 3:**
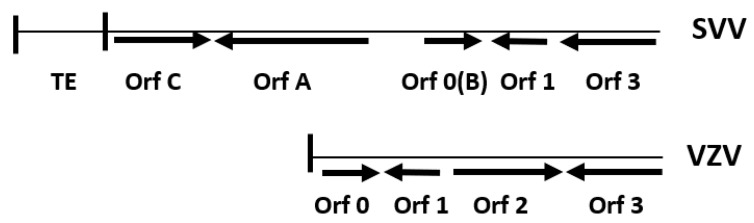
The left ends of the SVV and VZV genomes. SVV and VZV ORFs are designated by arrows indicating the site on each DNA strand. SVV DNA includes a terminal element (TE) but does not have an ORF 2. VZV DNA does not include a TE, ORF A, or ORF C. Vertical lines indicate TRL sequences.

The major differences in genetic content of SVV and VZV DNA occur at the left ends of the viral genomes (Figure 3). SVV DNA does not include a homologous gene to the VZV ORF 2, which is expressed as a 31 kD phosphoprotein within membranes of VZV infected cells but not within viral particles [18]. The VZV ORF 2 is nonessential, as deletion mutants replicate efficiently in cell culture and establish latency in ganglia of latently infected cotton rats.

The SVV left end includes an ORF A that is not found within the VZV genome [19]. The SVV ORF A is a truncated paralog of the SVV ORF 4 and ortholog of VZV ORF 4. The VZV ORF 4 is essential for in vitro replication and encodes an immediate early (IE) protein that transactivates VZV promoters [20,21,22]. Based upon extensive homology to VZV ORF 4, the SVV ORF 4 is also proposed as a viral transactivator protein. However, in an experimental study, the SVV ORF A did not transactivate SVV promoters [23]. This lack of transactivation may be due to the truncated SVV ORF A lacking the highly acidic amino-terminus and nuclear localization signal that is critical for VZV ORF 4 transactivation of reporter promoters [22]. The SVV ORF A promoter is stimulated by the SVV IE ORF 62 protein, and the 1.0 kb transcript is expressed in infected Vero cells [23]. An SVV ORF A deletion mutant replicates efficiently in Vero cells, indicating the gene is not essential for in vitro replication [23]. However, the ORF A is conserved within various SVV isolates and is expressed in tissues of SVV-infected monkeys, indicating its likely importance in viral pathogenesis [19].

The SVV ORF B, as originally identified on the SVV genome, is now known to be a homolog of the VZV ORF 0 (or ORF S/L), sharing 35% amino acid identity [19]. The VZV ORF 0 is essential for efficient replication in cell culture and in vivo and encodes a transmembrane protein that may play a role in viral DNA cleavage and packaging of concatemeric VZV DNA [20,24].

**Figure 4:**
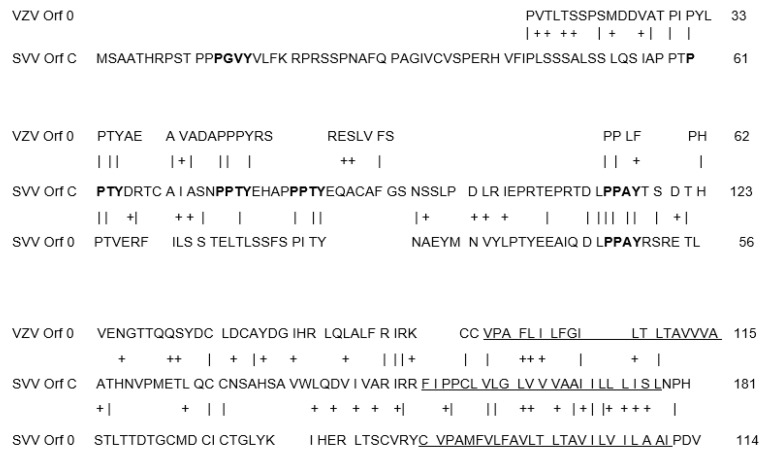
Amino acid alignment of the C-terminal region of the SVV and VZV UL56 homologs including SVV ORFs C and 0 (B) and VZV ORF 0. Vertical lines indicate amino acid identity, and + indicates amino acid similarity. PPRX motifs are in bold. Predicted transmembrane domains are underlined.

The SVV ORF C is a more recently identified gene located at the extreme left terminus of the SVV genome [25]. The SVV ORF C is a duplicated paralog of the SVV ORF 0 and encodes a 123 amino acid putative transmembrane protein. While the VZV genome does not have a corresponding gene to the SVV ORF C, it does have the homologous VZV ORF 0 [24]. The homology between the SVV ORF C, SVV ORF 0, and VZV ORF 0 is concentrated within the protein central region and the carboxy terminal hydrophobic domain (Figure 4).

The SVV ORF C, SVV ORF 0, and VZV ORF 0 are orthologs of the herpes simplex virus (HSV) unique long ORF 56 (UL56) gene family, which is conserved among alphaherpesviruses [25]. These UL56 orthologs include PPXY motifs, which are a consensus sequence for interaction with tryptophan-tryptophan (W-W) domains of cellular ubiquitin ligases that promote the vacuolar protein sorting pathway to induce budding of enveloped viruses from infected cells [26,27].

## 4. Transcription of the SVV and VZV Genomes

Initial transcript mapping of the SVV and VZV genomes during lytic infection employed Northern blot analysis and labeled restriction endonuclease DNA fragment probes [28,29]. These studies revealed similarities in SVV and VZV transcription profiles and confirmed that all regions of the viral genome are transcriptionally active during acute infection in cell culture. Subsequent gene array analyses confirmed transcription of each SVV and VZV ORF during in vitro infection [30,31].

Most recently, RNA sequencing approaches (RNA-Seq, dRNA-Seq) have provided comprehensive analyses of the SVV and VZV transcriptomes [32,33]. The transcriptional start sites (TSS) and cleavage and polyadenylation sites (CPAS) for each of the SVV and VZV protein coding genes have been identified. A complex transcriptional pattern has revealed alternative TSS usage, read-through transcription, and RNA splicing and the existence of various transcript isoforms for individual viral ORFs. While the transcriptional profiles for the SVV and VZV genomes were found to be quite similar, differences in the number and sizes of transcript isomers for specific SVV and VZV genes were identified.

## 5. Regulation of Gene Expression of the SVV and VZV Genomes

Similar to other herpesviruses, the SVV and VZV genomes are coordinately expressed into kinetic stages. While the cell-associated nature of SVV and VZV complicates the generation of high-titer viral stocks needed for synchronous in vitro infection, studies confirm that the viruses express IE, early, and late gene products.

IE gene expression does not require prior viral protein synthesis and generates regulatory proteins that transactivate the promoters of viral genes. SVV and VZV DNAs have been predicted to express four IE genes, ORFs 4, 61, 62, and 63, based upon homology to the HSV-1 IE genes. The IE62, encoded by ORFs 62 and 71, is considered as the major viral transactivator of IE, early, and late gene promoters [34]. The SVV IE62 transactivates SVV ORF 28/29 early gene expression by interacting with a 16 bp palindrome sequence within the ORF 28/29 early promoter that includes a cellular upstream stimulatory factor-binding site (USF) [35]. The VZV IE62 also interacts with viral promoters and transcription factors to transactivate viral gene expression [34,36].

The SVV and VZV ORF 61 encode a homolog to the HSV-1 ICP0 transactivator protein. The SVV IE61 transactivates its own promoter as well as IE, early, and late gene promoters [37]. The protein includes a ring finger motif and a nuclear localization sequence that is required for transactivation. The VZV IE61 also has transactivation properties, which are dependent on cell type and transfection conditions [38]. The SVV and VZV ORF 61 are dispensable for viral replication in cell culture [37,39].

Studies of the SVV and VZV ORF 63 gene products have shown differential effects on the regulation of viral promoters. While the SVV 63 protein did not transactivate the ORF 21 early gene promoter by itself, the SVV 63 upregulated SVV 62 transactivation of the ORF 21 promoter in neuronal cells [40]. In contrast, the SVV 63 down-regulated SVV 62 transactivation of the ORF 21 promoter in Vero and Mewo cells. The SVV ORF 63 is not essential for in vitro replication, but its expression stimulates viral growth [41]. The VZV ORF 63 protein represses ORF 62 IE transcription, activates the thymidine kinase early gene expression, and has no effect on expression of late glycoprotein genes [42].

The SVV ORF 4 protein only weakly transactivated an early promoter but stimulated IE62 transactivation of the same promoter [23]. As indicated above, the SVV ORF A truncated protein homolog of ORF 4 does not transactivate SVV promoters [23]. The VZV ORF 4 is an IE gene that is essential for viral replication, can transactivate viral gene expression, and can augment IE62 stimulation of viral promoters [21,36].

Herpesvirus early gene products include enzymes involved in DNA synthesis. Several SVV and VZV early genes have been characterized including the deoxyuridine nucleotidohydrolase (dUTPase, ORF 8), uracil glycosylase (ORF 59), and thymidine kinase (ORF 36), which express enzymes involved in viral DNA replication and repair [43,44,45,46].

Herpesvirus late genes encode structural proteins including the viral capsid and envelope glycoproteins. Several VZV ORFs encode proteins involved in the structure and assembly process of viral capsid formation [47]. While SVV capsid assembly has not been analyzed, the SVV and VZV major capsid proteins encoded by ORF 40 are similar in size (156 kD) and share over 70% amino acid identity. SVV and VZV express at least nine glycoproteins, including gB, gC, gE, gH, gI, gK, gL, gN, and gM, which play a critical role in viral attachment, penetration, and cell-to-cell spread [48,49,50]. The SVV and VZV genomes do not encode a homolog to the HSV-1 glycoprotein D.

The development of RNA-Seq and dRNA-Seq methodology has permitted low titer, cell-free virus synchronous infection to analyze the kinetics of VZV transcription and gene expression [33]. VZV-infected culture cells were infected in the presence of cycloheximide (CHX) or phosphonoacetic acid (PAA) to identify IE or early transcripts, respectively. Late mRNAs were identified later in infection without metabolic inhibitors. VZV ORFs 4, 61, and 63 were highly expressed as IE genes in CHX-infected cells. ORF 0 was also expressed as an IE gene. Interestingly, VZV ORF 62 was not expressed under IE conditions but rather expressed late during infection, suggesting that a virion tegument derived ORF 62 protein is responsible for transactivation of IE transcription. Early gene expression was dependent on IE proteins, and 53 early transcripts were identified. Late gene expression included 28 leaky late (LL) and 41 true late (TL) transcripts, which were expressed at low levels prior to DNA synthesis or after DNA replication, respectively. To date, a similar kinetic analysis for SVV gene expression has not been reported.

## 6. Expression of SVV and VZV Viral Latency Associated Transcripts (VLTs)

Following primary varicella infection, SVV and VZV establish life-long latency within cells of neural ganglia [1,51]. Induction of viral reactivation may occur to cause secondary disease, such as herpes zoster [4]. An understanding of the molecular basis of viral latency and reactivation is needed for development of antiviral strategies against herpes zoster and postherpetic neuralgia.

**Figure 5:**
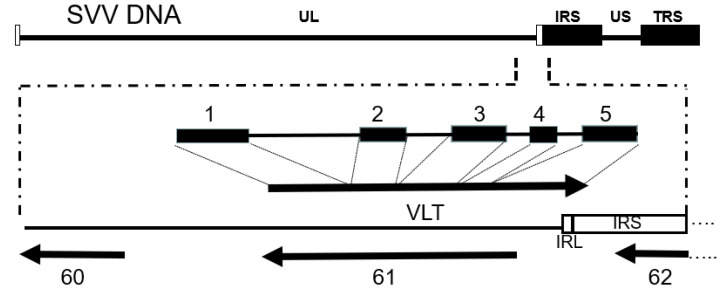
The SVV latency associated transcript (VLT) is a spliced RNA consisting of five exons (blocks) and introns (lines) mapping to the UL/IRS junction of SVV DNA and antisense to ORF 61. Multiple other isoforms are also transcribed from this locus during lytic infection. From Braspenning et al. [32].

SVV gene expression in neural ganglia of latently infected monkeys is restricted to a viral latency-associated transcript (VLT) [52]. This transcript was initially detected at low abundance by reverse-transcriptase PCR (RT-PCR) in trigeminal, cervical, and lumbar neural ganglia but not in lung or liver tissues during SVV latency. The SVV VLT was localized to the genomic UL/IRS junction and oriented antisense to the SVV ORF 61 (HSV-1 ICP0 homolog), which encodes an IE transactivator protein (Figure 5).

Subsequently, RNA-Seq was employed to reveal a VZV VLT in human trigeminal ganglia [53]. The VZV VLT, like the SVV VLT, was determined to map antisense to the viral ORF 61. While multiple spliced VLT isoforms were generated during lytic infection, a single VLT isoform was detected in latently infected ganglia. The VLT was associated with a 136 amino acid protein expressed with late kinetics in cultured cells and in skin biopsy derived from a shingles patient but not in human ganglia.

RNA-Seq analysis has also provided a detailed characterization of the SVV VLT [32]. Similar to the VZV VLT, during lytic infection of culture cells, the SVV VLT locus is expressed as multiple spliced transcript isoforms with alternative transcription start sites. Some of these isoforms are predicted to be translated as peptides with homology to the VZV VLT protein. However, in ganglia derived from latently infected monkeys, a single spliced VLT isoform was detected at low abundance. This VLT was detected within neurons but not non-neuronal cells of latently infected ganglia [32].

Expression of the SVV and VZV VLT oriented antisense to the ORF 61 and HSV-1 ICP0 homolog is consistent with gene expression during latency of other alphaherpesviruses [52]. HSV-1, HSV-2, EHV-1, EHV-4, PRV, bovine herpesvirus 1 (BHV-1), and feline herpesvirus 1 (FHV-1) each express a transcript antisense to an ICP0 homolog in neural ganglia of latently infected animals [32].

The role of the SVV and VZV VLT in viral latency and reactivation is not understood. A SVV ORF 61 deletion mutant, with a corresponding disrupted VLT, produced clinical varicella in rhesus macaques and established latent infection in neural ganglia [54]. In addition, a VZV ORF 61 deletion mutant was able to establish latent infection in neural ganglia of infected cotton rats [39]. These results indicate that the SVV and VZV VLT is not essential for the establishment of viral latency. It is possible that the VLT may play a role in maintenance of the latent state or induction of reactivation by binding of the VLT to the complementary ORF 61 promoter and inhibiting IE61 expression and transactivation of other viral promoters. Such a hypothesis is contradicted by studies demonstrating that HSV-1 reactivation in mice is not mediated by VLT antisense regulation of the ICP0 gene [55]. Finally, the SVV and VZV VLT may promote neuronal cell survival and enhance viral latency by inhibiting apoptosis, as has been proposed for HSV-1 and BHV-1 VLT [56,57]. Hopefully, further studies will elucidate the mechanisms by which the SVV and VZV VLT promote viral latency and/or reactivation.

## 7. Evolutionary Relationship between SVV and VZV

**Figure 6:**
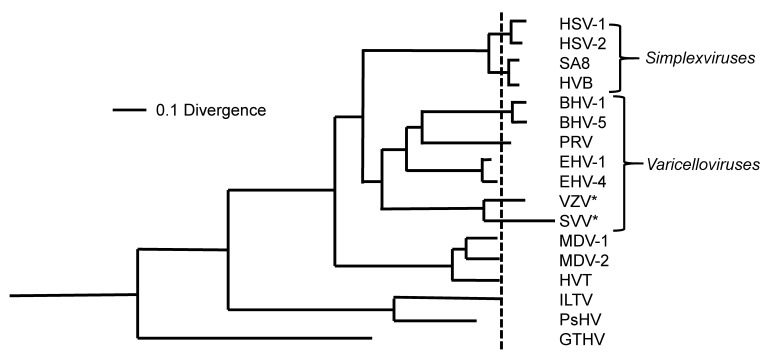
Phylogenetic tree of the alphaherpesviruses constructed based on amino acid alignment of six conserved herpesvirus genes [59]. SVV and VZV are denoted with an asterisk. Abbreviations: MDV-1, MDV-2, Marek’s disease virus; HVT, herpesvirus of turkeys; ILTV, infectious laryngotracheitis virus; PsHV, psittacid herpesvirus 1; GTHV, green turtle herpesvirus. Other abbreviations are indicated in the text. Reprinted from *Virus Research* [59] with permission from the publisher.

A primordial herpesvirus infecting ancestral vertebrates gave rise to the *Herpesviridae* family an estimated 400 million years ago (mya) [58,59]. Figure 6 shows a phylogenetic tree of the *Alphaherpesvirinae* assembled based upon alignment of amino acid sequences for six conserved genes among alpha-, beta-, and gammaherpesviruses [59]. Based upon this analysis, the alphaherpesviruses diverged from the beta and gamma herpesviruses around 190 to 200 mya [59]. Then, around 70 mya, the alphaherpesviruses evolved into the *simplexvirus* (ultimately HSV-1, HSV-1, herpesvirus B (HVB), and simian agent 8 (SA8)), and *varicellovirus* (VZV, EHV-1, EHV-1 PRV, FHV-2) genera [58].

An ancestral varicella virus may have arisen in an early African primate around 70 mya [58]. Fossil and phylogenetic evidence indicate that cercopithecoid monkeys and hominoids (great apes) diverged around 25 to 30 mya. Based upon the hypothesis of cospeciation, SVV and VZV likely evolved from this ancestral varicella virus, which diverged as closely related, species-specific viruses for Old World monkeys or humans, respectively.

VZV evolution continues as evident by the current existence of five major phylogeographical clades, which are differentiated by single-nucleotide polymorphisms (SNP) [60]. Clades 1, 3, and 4 include VZV isolates predominately derived from Europe and North America. Clade 2 and clade 5 include VZV isolates of Asian and Indian origin, respectively. While the clades are distinguished by specific SNPs, each of the VZV isolates share at least 99.8% DNA sequence identity. The clades represent clusters of VZV strains presumed to have evolved from a common ancestor.

The SVV deltaherpesvirus (DHV) is currently the only SVV isolate that has been completely sequenced and annotated [10]. DHV was derived from an infected patas monkey during a 1973 outbreak at a primate facility in Louisiana, U.S.A. GenBank also includes an unannotated DNA sequence of SVV isolated from an infected African Green monkey in China. Additional SVV isolates have been derived from various species of Old World monkeys and from outbreaks at various geographical locations [5]. Future genetic analyses of these SVV isolates will reveal further insight regarding the diversity of SVV isolates and the coevolution of SVV and VZV.

## Figures and Tables

**Table 1:** Comparison of the SVV and VZV genomes.

Size (Base Pairs)			G + C %		
	SVV ^1^	VZV ^2^		SVV ^1^	VZV ^2^
TRS/IRS	7557	7319.5		65.0%	59.0%
US	4904	5232		39.1%	42.8%
Total S	20,018	19,871		58.6%	54.7%
TRL/IRL	65	88.5		69.3%	68.4%
UL	104,036	104,836		38.3%	44.3%
TE ^3^	666	-		53.8%	-
Total L	104,767	105,013		38.3%	44.3%
Total Genome	124,785	124,884		40.4%	46.0%

^1^ data from Gray et al. (2001) [10]; ^2^ data derived from Davison and Scott (1986) [11]; ^3^ terminal element-SVV genome left end.

## Supplementary Materials

The following are available online at https://www.mdpi.com/article/10.3390/v14050844/s1, Table S1. Comparison of the SVV and VZV genomes.
