# Supplementary material for: Comparative Analysis of the Simian Varicella Virus and Varicella Zoster Virus Genomes
Source: Viruses. 2022 Apr 19;14(5):844. doi: 10.3390/v14050844 (PMC9144398; doi:10.3390/v14050844)
Supplement: Supplementary file 1 [file viruses-14-00844-s001.zip › viruses-1679277-supplementary.pdf]

Supplemental Table S1. SVV and VZV open reading frames.

ORF[1]	SVV Size (aa)	SVV Size(kDa)[2]	SVV/ VZV % homology[3]	VZV Size (aa)[2]	HSV-1 homolog	Putative Function[4]/Notes
C	123	13.5	----	----	UL56	Paralog of ORF 0 (B), Membrane protein
A	293	33.5	----	----	UL54	Truncated paralog of ORF 4
0 (B)	114	12.7	29.9	157-224	UL56	DNA cleavage and packaging
1	101	11.7	27.3	108	None	Membrane protein
2	----	----	----	238	None	VZV membrane protein, no SVV homolog
3	183	19.7	63.7	179	UL55	Virion assembly
4	470	54.3	43.2	452	UL54	Transcriptional activator, immediate early protein
5	337	42.0	59.3	340	UL53	Glycoprotein K
6	1081	123.3	37.7	1083	UL52	Component of DNA helicase-primase complex
7	231	25.3	72.9	259	UL51	Virion phosphoprotein
8	395	44.9	38.2	396	UL50	DeoxyUTPase
9A	87	9.7	68.8	87	UL49A	Glycoprotein N
9	301	33.2	59.4	302	UL49	Tegument protein
10	406	46.6	62.5	410	UL48	Transcriptional activator, tegument protein
11	642	71.7	50.8	819	UL47	Tegument protein
12	655	73.4	60.1	661	UL46	Tegument protein
13	295	33.9	71.1	301	None	Thymidylate synthetase
14	540	60.6	43.8	560	UL44	Glycoprotein C
15	421	47.3	36.2	406	UL43	Membrane protein
16	385	42.8	46.8	408	UL42	Associated with DNA polymerase
17	471	53.6	55.0	455	UL41	Host shutoff virion protein
18	311	36.2	72.6	306	UL40	Ribonucleotide reductase, small subunit
19	783	88.2	68.7	775	UL39	Ribonucleotide reductase, large subunit
20	467	52.9	60.5	483	UL38	Capsid protein
21	1041	116.8	52.0	1038	UL37	Tegument protein
22	2653	294.8	48.9	2763	UL36	Tegument protein

23	228	23.8	47.3	235	UL35	Capsid protein
24	214	23.9	61.5	269	UL34	Membrane phosphoprotein
25	153	17.4	45.2	156	UL33	Viral DNA cleavage/packaging
26	572	65.1	61.3	585	UL32	DNA cleavage/packaging
27	310	35.4	74.4	333	UL31	Nuclear phosphoprotein
28	1172	113.2	65.7	1194	UL30	DNA polymerase
29	1194	132.0	71.9	1204	UL29	Single-stranded DNA binding protein
30	760	86.5	61.3	770	UL28	Viral DNA cleavage/packaging
31	916	104.0	75.4	868	UL27	Glycoprotein B
32	135	15.0	49.6	143	None	Phosphoprotein
33	588	65.1	64.4	605	UL26	Protease, capsid assembly protein
34	579	65.7	61.1	579	UL25	Viral DNA cleavage/packaging
35	248	28.7	49.7	258	UL24	Membrane protein
36	337	37.9	52.3	341	UL23	Thymidine kinase
37	852	96.8	55.5	841	UL22	Glycoprotein H
38	533	59.8	60.3	541	UL21	Virion protein
39	223	25.4	53.1	240	UL20	Envelope protein, viral egress
40	1392	155.9	73.3	1396	UL19	Major capsid protein
41	315	34.3	70.5	316	UL18	Capsid protein
42/45	744	84.1	67.7	747	UL15	Spliced product[5]<br>Viral terminase
43	678	75.9	47.3	676	UL17	Viral DNA cleavage/packaging
44	360	39.8	69.3	363	UL16	Virion protein
46	199	22.5	58.2	199	UL14	Tegument protein
47	507	57.7	64.8	510	UL13	Protein Kinase
48	509	57.8	56.2	551	UL12	Deoxyribonuclease
49	82	9.2	50.0	81	UL11	Myristylated virion protein
50	439	49.5	57.5	435	UL10	Glycoprotein M

51	816	92.8	53.7	835	UL9	Origin binding protein
52	765	86.0	50.4	771	UL8	Component of DNA helicase-primase complex
53	304	34.5	56.6	331	UL7	Cytoplasmic envelope protein, viral egress
54	735	83.5	59.5	769	UL6	Viral DNA cleavage/packaging
55	869	97.8	75.2	881	UL5	Component of DNA helicase-primase complex
56	187	21.3	37.7	244	UL4	Nuclear protein
57	72	8.2	39.5	71	None	Nonessential VZV protein
58	203	22.9	42.0	221	UL3	Phosphoprotein
59	300	34.6	55.4	305	UL2	Uracil DNA glycosylase
60	175	20.2	43.5	159	UL1	Glycoprotein L
61	503	54.1	42.8	467	RL2	Transcriptional activator, repressor, immediate early protein
62	1279	136.8	58	1310	RS1	Transcriptional activator, immediate early protein
63	261	29.3	52	278	US1	Transcriptional activator, Immediate early protein
64	187	21.1	56	180	US10	Tegument phosphoprotein
65	77	9.0	49	102	US9	Tegument phosphoprotein
66	345	38.9	66	393	US3	Protein Kinase
67	353	40.5	37	354	US7	Glycoprotein I
68	604	67.6	47	623	US8	Glycoprotein E
69	187	21.1	56	180	US10	Duplicate of ORF 64
70	261	29.3	52	278	US1	Duplicate of ORF 63
71	1279	136.8	58	1310	ICP4	Duplicate of ORF 62

[1] SVV ORFs derived from *Gray et al.* (2001) [10]. VZV ORFs derived from *Davison and Scott* (1986) [11].
[2] Predicted size based on amino acid sequence.
[3] Based on % amino acid identity between homologous SVV and VZV proteins
[4] Based on known function or function of HSV-1 homolog
[5] Predicted spliced gene including ORF 42 and 45 exons
